# A new polymorph of *N*-phenyl­phthalimide

**DOI:** 10.1107/S1600536809006746

**Published:** 2009-02-28

**Authors:** L. Yu. Izotova, J. M. Ashurov, B. T. Ibragimov, E. Weber

**Affiliations:** a Institute of Bioorganic Chemistry, Academy of Sciences of Uzbekistan, H. Abdullaev Str. 83, Tashkent 100125, Uzbekistan; b Institut für Organische Chemie, TU, Bergakademie Freiberg, Leipziger Strasse 29, D09596 Freiberg/Sachsen, Germany

## Abstract

During an attempt to prepare a cocrystal of *N*-phenyl­phthalimide, C_14_H_9_NO_2_, with *N*-(2,3,4,5,6-penta­fluoro­phen­yl)phthalimide, a new ortho­rhom­bic polymorph of the first component was obtained. This new form has *Z*′ = 0.5 and the mol­ecule is located around a twofold axis, whereas in the previously reported polymorph (space group *Pbca*), the mol­ecule has no crystallographically imposed symmetry. Pairs of C—H⋯O inter­actions between inversion-related phthalimide units arrange mol­ecules into tapes that are further assembled into (010) layers *via* stacking inter­actions between phthalimide fragments [inter­planar distance = 3.37 (5) Å].

## Related literature

For the crystal structure of another polymorph of *N*-phenyl­phthalimide, see: Magomedova *et al.* (1981 ▶); Schwarzer & Weber (2008 ▶).
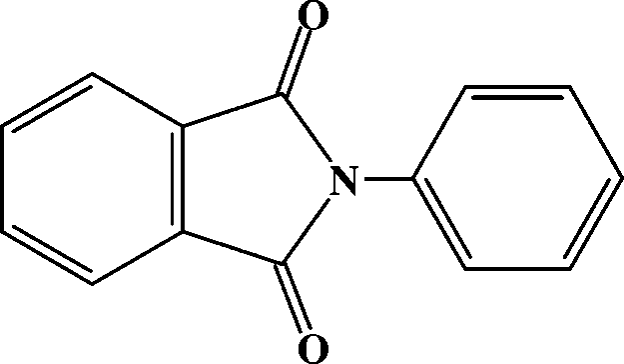


## Experimental

### 

#### Crystal data

C_14_H_9_NO_2_
*M* *_r_* = 223.22
Orthorhombic, 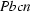

*a* = 5.5480 (11) Å
*b* = 23.801 (5) Å
*c* = 8.0250 (16) Å
*V* = 1059.7 (4) Å^3^
*Z* = 4
Mo *K*α radiation
μ = 0.10 mm^−1^
*T* = 293 K
0.2 × 0.05 × 0.05 mm

#### Data collection

Stoe Stadi-4 diffractometer
Absorption correction: none
1039 measured reflections
1039 independent reflections
662 reflections with *I* > 2σ(*I*)
3 standard reflections every 60 reflections intensity decay: 3.9%

#### Refinement

*R*[*F* ^2^ > 2σ(*F* ^2^)] = 0.067
*wR*(*F* ^2^) = 0.137
*S* = 1.17
1039 reflections
80 parameters
H-atom parameters constrained
Δρ_max_ = 0.17 e Å^−3^
Δρ_min_ = −0.15 e Å^−3^

### 

Data collection: *STADI4* (Stoe & Cie, 1997 ▶); cell refinement: *STADI4*; data reduction: *X-RED* (Stoe &Cie, 1997 ▶); program(s) used to solve structure: *SHELXS97* (Sheldrick, 2008 ▶); program(s) used to refine structure: *SHELXL97* (Sheldrick, 2008 ▶); molecular graphics: *XP* (Siemens, 1994 ▶); software used to prepare material for publication: *SHELXL97*.

## Supplementary Material

Crystal structure: contains datablocks I, global. DOI: 10.1107/S1600536809006746/gk2189sup1.cif

Structure factors: contains datablocks I. DOI: 10.1107/S1600536809006746/gk2189Isup2.hkl

Additional supplementary materials:  crystallographic information; 3D view; checkCIF report

## Figures and Tables

**Fig. 1.**
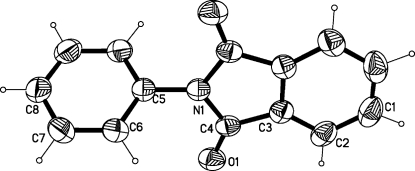
Perspective view of the molecule showing 40% probability displacement ellipsoids for the non-hydrogen atoms.

**Fig. 2.**
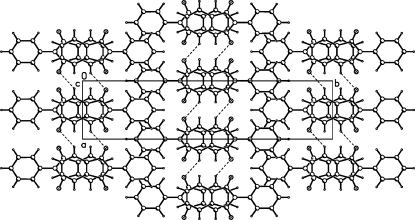
Packing diagram for polymorph II; viewed down the c axis. Hydrogen bonds are shown as dashed lines.

## supplementary crystallographic information

### Comment

The crystal structure of the polymorph I of the title compound has previously been reported [Magomedova *et al.*, 1981; Schwarzer & Weber, 2008]. In the course of our studies on the crystal-engineering behaviour of *N*-aryl substituted phthalimides (Schwarzer & Weber, 2008) and on polymorphism in general a new polymorph of *N*-phenylphthalimide (designated as II), was obtained. The molecular structure of *N*-phenylphthalimide (Fig. 1) is similar to that in the polymorph I, exept the twist angle between the phenyl and phthalimide units which is larger in polymorph II [64.09 (10)°] than in polymorph I [56.73 (4)°] owing to differences in the crystal packing and intermolecular interactions. The most prominent interactions in the polymorph I are carbonyl-carbonyl interaction leading to a short C1=O1···C2=O2 contact (3.08 Å) and a weak C3—H1···O1 interaction (2.65 Å, 137°). In the form II molecules of *N*-phenylphthalimide related by inversion interact *via* C2—H2···O1^i^ hydrogen bonds (2.66 Å, 145°; symmetry code: (i) 1 - *x*, 1 - *y*, -*z*) forming zigzag tapes parallel to the crystallographic (102) plane (Fig. 2). These tapes are arranged into (010) layers *via* stacking interactions between phthalimide units. Within the stacks, the distance between planes of phthalimide units of consecutive molecules is 3.37 (5) Å.

### Experimental

*N*-Phenylphthalimide was synthesized from aniline and phthalic anhydride according to the procedure described by Schwarzer & Weber (2008). After evaporation of solvent from an acetone solution containing equimolar mixture of *N*-phenylphthalimide and *N*-(2,3,4,5,6,-pentafluorophenyl)phthalimide two types of crystals have deposited. The crystals of the plate form (m.p. 456.5 K) appeared to be the polymorph I of *N*-phenylphthalimide whereas bulky needles the polymorph II (m.p. 485 K). Both types of crystals were stable in the air.

### Refinement

All H-atoms were positioned geometrically (C—H 0.93 Å) and refined as riding on their carrier atoms with *U*_iso_(H) = 1.2*U*_eq_(C).

### Crystal data

**Table:**

C_14_H_9_NO_2_	*F*(000) = 464
*M**_r_* = 223.22	*D*_x_ = 1.399 Mg m^−^^3^
Orthorhombic, *P**b**c**n*	Mo *K*α radiation, λ = 0.71073 Å
Hall symbol: -P 2n 2ab	Cell parameters from 25 reflections
*a* = 5.5480 (11) Å	θ = 10–25°
*b* = 23.801 (5) Å	µ = 0.10 mm^−^^1^
*c* = 8.0250 (16) Å	*T* = 293 K
*V* = 1059.7 (4) Å^3^	Needle, colourless
*Z* = 4	0.2 × 0.05 × 0.05 mm

### Data collection

**Table:**

Stoe Stadi-4 diffractometer	*R*_int_ = 0.0000
Radiation source: fine-focus sealed tube	θ_max_ = 26.0°, θ_min_ = 1.7°
graphite	*h* = 0→6
ω–2τ scans	*k* = −29→0
1039 measured reflections	*l* = 0→9
1039 independent reflections	3 standard reflections every 60 reflections
662 reflections with *I* > 2σ(*I*)	intensity decay: 3.9%

### Refinement

**Table:**

Refinement on *F*^2^	Secondary atom site location: difference Fourier map
Least-squares matrix: full	Hydrogen site location: inferred from neighbouring sites
*R*[*F*^2^ > 2σ(*F*^2^)] = 0.067	H-atom parameters constrained
*wR*(*F*^2^) = 0.137	*w* = 1/[σ^2^(*F*_o_^2^) + (0.0266*P*)^2^ + 0.8879*P*] where *P* = (*F*_o_^2^ + 2*F*_c_^2^)/3
*S* = 1.17	(Δ/σ)_max_ < 0.001
1039 reflections	Δρ_max_ = 0.17 e Å^−^^3^
80 parameters	Δρ_min_ = −0.15 e Å^−^^3^
0 restraints	Extinction correction: *SHELXL97* (Sheldrick, 2008), Fc^*^=kFc[1+0.001xFc^2^λ^3^/sin(2θ)]^-1/4^
Primary atom site location: structure-invariant direct methods	Extinction coefficient: 0.0123 (18)

### Special details

**Table:**

Geometry. All e.s.d.'s (except the e.s.d. in the dihedral angle between two l.s. planes) are estimated using the full covariance matrix. The cell e.s.d.'s are taken into account individually in the estimation of e.s.d.'s in distances, angles and torsion angles; correlations between e.s.d.'s in cell parameters are only used when they are defined by crystal symmetry. An approximate (isotropic) treatment of cell e.s.d.'s is used for estimating e.s.d.'s involving l.s. planes.
Refinement. Refinement of *F*^2^ against ALL reflections. The weighted *R*-factor *wR* and goodness of fit *S* are based on *F*^2^, conventional *R*-factors *R* are based on *F*, with *F* set to zero for negative *F*^2^. The threshold expression of *F*^2^ > σ(*F*^2^) is used only for calculating *R*-factors(gt) *etc*. and is not relevant to the choice of reflections for refinement. *R*-factors based on *F*^2^ are statistically about twice as large as those based on *F*, and *R*- factors based on ALL data will be even larger.

### Fractional atomic coordinates and isotropic or equivalent isotropic displacement parameters (Å^2^)

**Table:**

	*x*	*y*	*z*	*U*_iso_*/*U*_eq_	
N1	0.0000	0.39051 (14)	0.2500	0.0475 (9)	
O1	0.3444 (4)	0.40588 (9)	0.0909 (3)	0.0624 (7)	
C1	0.1048 (7)	0.58237 (14)	0.2026 (4)	0.0740 (12)	
H1	0.1731	0.6164	0.1709	0.089*	
C2	0.2140 (6)	0.53244 (14)	0.1544 (4)	0.0626 (10)	
H2	0.3548	0.5324	0.0916	0.075*	
C3	0.1056 (5)	0.48304 (12)	0.2034 (3)	0.0491 (8)	
C4	0.1752 (6)	0.42378 (12)	0.1702 (4)	0.0479 (8)	
C5	0.0000	0.33042 (18)	0.2500	0.0498 (11)	
C6	0.1868 (6)	0.30175 (13)	0.3253 (4)	0.0601 (9)	
H6	0.3125	0.3213	0.3754	0.072*	
C7	0.1855 (6)	0.24375 (14)	0.3257 (5)	0.0765 (12)	
H7	0.3099	0.2241	0.3771	0.092*	
C8	0.0000	0.2150 (2)	0.2500	0.0820 (19)	
H8	0.0000	0.1759	0.2500	0.098*	

### Atomic displacement parameters (Å^2^)

**Table:**

	*U*^11^	*U*^22^	*U*^33^	*U*^12^	*U*^13^	*U*^23^
N1	0.044 (2)	0.049 (2)	0.049 (2)	0.000	0.0052 (18)	0.000
O1	0.0502 (13)	0.0738 (15)	0.0631 (15)	0.0017 (12)	0.0078 (12)	0.0049 (12)
C1	0.088 (3)	0.0545 (19)	0.079 (3)	−0.0123 (18)	−0.033 (2)	0.0080 (18)
C2	0.063 (2)	0.066 (2)	0.059 (2)	−0.0128 (18)	−0.0198 (18)	0.0059 (18)
C3	0.0502 (17)	0.0543 (18)	0.0427 (18)	−0.0033 (14)	−0.0134 (15)	0.0018 (14)
C4	0.0409 (16)	0.0589 (19)	0.0438 (17)	−0.0017 (16)	−0.0061 (15)	0.0036 (15)
C5	0.048 (2)	0.050 (3)	0.052 (3)	0.000	0.003 (2)	0.000
C6	0.0476 (18)	0.062 (2)	0.070 (2)	0.0027 (17)	−0.0001 (18)	0.0049 (18)
C7	0.057 (2)	0.067 (2)	0.106 (3)	0.013 (2)	0.011 (2)	0.018 (2)
C8	0.058 (3)	0.057 (3)	0.130 (5)	0.000	0.033 (4)	0.000

### Geometric parameters (Å, °)

**Table:**

N1—C4^i^	1.408 (3)	C3—C4	1.487 (4)
N1—C4	1.408 (3)	C5—C6	1.380 (4)
N1—C5	1.430 (5)	C5—C6^i^	1.380 (4)
O1—C4	1.211 (3)	C6—C7	1.380 (4)
C1—C2	1.389 (4)	C6—H6	0.9300
C1—C1^i^	1.389 (8)	C7—C8	1.378 (4)
C1—H1	0.9300	C7—H7	0.9300
C2—C3	1.378 (4)	C8—C7^i^	1.378 (4)
C2—H2	0.9300	C8—H8	0.9300
C3—C3^i^	1.389 (6)		
			
C4^i^—N1—C4	111.6 (4)	N1—C4—C3	105.8 (3)
C4^i^—N1—C5	124.22 (18)	C6—C5—C6^i^	120.7 (4)
C4—N1—C5	124.22 (18)	C6—C5—N1	119.6 (2)
C2—C1—C1^i^	121.2 (2)	C6^i^—C5—N1	119.6 (2)
C2—C1—H1	119.4	C7—C6—C5	119.5 (4)
C1^i^—C1—H1	119.4	C7—C6—H6	120.3
C3—C2—C1	117.4 (3)	C5—C6—H6	120.3
C3—C2—H2	121.3	C8—C7—C6	120.0 (4)
C1—C2—H2	121.3	C8—C7—H7	120.0
C2—C3—C3^i^	121.4 (2)	C6—C7—H7	120.0
C2—C3—C4	130.2 (3)	C7—C8—C7^i^	120.3 (5)
C3^i^—C3—C4	108.40 (16)	C7—C8—H8	119.8
O1—C4—N1	125.2 (3)	C7^i^—C8—H8	119.8
O1—C4—C3	129.0 (3)		

Symmetry codes: (i) −*x*, *y*, −*z*+1/2.
